# Sparstolonin B exerts beneficial effects on prostate cancer by acting on the reactive oxygen species‐mediated PI3K/AKT pathway

**DOI:** 10.1111/jcmm.16560

**Published:** 2021-05-05

**Authors:** Shaozhuang Liu, Jiapeng Hu, Changlong Shi, Li Sun, Wentao Yan, Yongsheng Song

**Affiliations:** ^1^ Department of Urology Shengjing Hospital of China Medical University Shenyang China; ^2^ Department of Pediatrics Shengjing Hospital of China Medical University Shenyang China; ^3^ Department of General Surgery Shengjing Hospital of China Medical University Shenyang China; ^4^ Department of Urology The Fifth People's Hospital of Fudan University Shanghai China

**Keywords:** apoptosis, oxidative stress, proliferation, prostate cancer, sparstolonin B

## Abstract

Prostate cancer is a major health concern in males worldwide, owing to its high incidence. Sparstolonin B (SsnB), a component of the Chinese herbal medicine *Sparganium stoloniferum*, is used to treat many diseases. However, the effects and mechanisms of action of SsnB in prostate cancer have not yet been reported. In this study, we evaluated the effects of SsnB on cellular processes and tumour growth. In particular, we verified that SsnB could inhibit the proliferation, migration and invasion of prostate cancer cells and induce apoptosis by activating G2/M phase arrest in vitro based on a series of cytological experiments. In vivo, we found that SsnB could inhibit tumour growth in nude mouse xenograft models. We further confirmed that SsnB could repress the PI3K/AKT pathway by increasing reactive oxygen species (ROS) accumulation and oxidative stress. Collectively, SsnB inhibits tumour growth and induces apoptosis in prostate cancer via the suppression of the ROS‐mediated PI3K/AKT pathway and may be a new alternative to adjuvant therapy for prostate cancer.

## 1 INTRODUCTION

Prostate cancer (PCa) is a major health concern worldwide, owing to its increasing morbidity and mortality. Among tumours affecting males, the incidence rate of PCa ranks 1st, 2nd and 7th in the United States, Europe and China, respectively, and it has the 2nd and 3rd highest death rates among cancers in the United States and Europe, respectively.^1^, ^2^, ^3^ A wide range of risk factors for PCa have been identified, including family history, hormone levels, ethnicity, ageing, oxidative stress, sexually transmitted diseases, diet, smoking, environmental agents, occupation and sexual activity.^4^, ^5^ However, the mechanism underlying PCa progression is not clear.^6^ Current therapeutic regimens of PCa mainly include radical therapy, chemotherapy and endocrine therapy,^7^ all of which have various side effects. Accordingly, an effective and safe new strategy for the treatment of PCa is urgently needed.^8^

**FIGURE 1:**
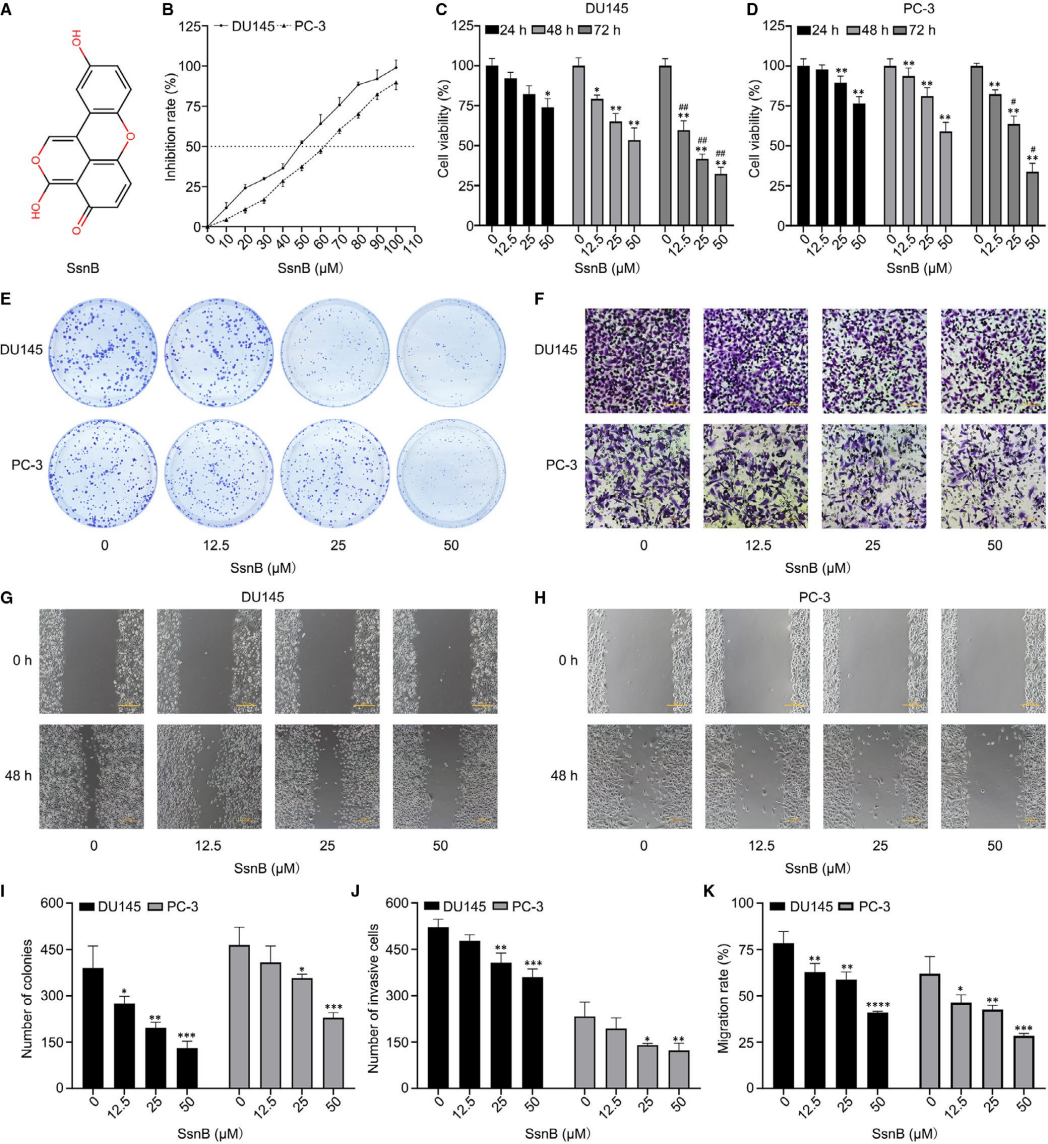
Sparstolonin B (SsnB) inhibits the proliferation, migration and invasion of prostate cancer (PCa) cells in vitro. A, Chemical structure of SsnB. B, Inhibition rates for various concentrations of SsnB on DU145 and PC‐3 cells over 48 h. C, D Cell viabilities of DU145 and PC‐3 after 24, 48 and 72 h of treatment. E, I Colony formation abilities of DU145 and PC‐3, as detected by the colony formation assay. F, J Invasion abilities of DU145 and PC‐3, as detected by the Transwell assay (scale bar: 50 μm). G, H, K Migration abilities of DU145 and PC‐3, as detected by the wound healing assay (scale bar: 100 μm). *P < .05, **P < .01, ***P < .001, ****P < .0001 vs the control group; ^#^ *P* <.05, ^##^ *P* <.01 vs groups treated with the same concentration at 24 h

*Sparganium stoloniferum*, an aquatic herb found in North and East China, is used to regulate menstruation and to promote galactosis and spasmolysis in traditional Chinese medicine (TCM).^9^ Sparstolonin B (SsnB) has been extracted from the rhizomes of this plant. Both X‐ray crystallography and nuclear magnetic resonance spectroscopy have revealed that SsnB, whose structural formula is C_15_H_8_O_4_, is a polyphenol (Figure 1A) containing two core components of xanthone and isocoumarin.^10^ Xanthones have established antioxidant, anti‐inflammatory, immunomodulatory, anti‐tumour, antimicrobial, anticholinesterase and anticonvulsant functions.^11^, ^12^, ^13^, ^14^ Similarly, Isocoumarins have anti‐tumour, anticoagulant, anti‐diabetes and antimicrobial bioactivities.^15^, ^16^ The special structure of SsnB makes it promising for the treatment of many diseases, especially inflammatory diseases, neurological diseases and tumours.^9^

The relationship between SsnB and the progression of PCa has thus far not been explored, and the mechanism underlying its anti‐tumour effect is still unclear. In this study, we evaluated the functions of SsnB in PCa with respect to proliferation, migration, invasion, apoptosis, cell cycle progression and oxidative stress, both in vitro and in vivo, for the first time, providing a basis for the development of novel comprehensive therapies for PCa.

## 2 MATERIALS AND METHODS

### 2.1 Cell culture

The PCa cell lines DU145 (Cell Bank of the Chinese Academy of Sciences) and PC‐3 (China Center for Type Culture Collection) were obtained. DU145 and PC‐3 cells were cultured in RPMI 1640 (HyClone) and Ham's F‐12K (Kaighn's) (GIBCO) medium, respectively, containing 10% (V/V) superfine foetal bovine serum (FBS) (Bioind, Beit HaEmek) and 1% (V/V) penicillin‐streptomycin (5,000 U/mL) solution (GIBCO). The medium was changed every 2 days, and 0.25% trypsin‐EDTA (GIBCO) was used for dissociation when the cell density reached 80%. Both cell lines were maintained in a constant humidified incubator with 5% (V/V) CO_2_ at 37°C. The stock solution of SsnB (Sigma‐Aldrich Corp.) (Figure 1A) was prepared in dimethyl sulphoxide (DMSO) (Solarbio). Cells were exposed to SsnB in fresh medium at different doses, and an equal volume of DMSO solution was used for the vehicle control group.

### 2.2 Cell Counting Kit‐8 assay

A Cell Counting Kit‐8 (CCK‐8) assay (APExBIO) was used to evaluate the effects of different concentrations of SsnB on proliferation in PCa cell lines. The cells were seeded into 96‐well plates at a density of 3 × 10^3^ cells per well for 24 hours, and then SsnB at different concentrations from 0 µmol/L to 100 µmol/L was replenished in each well for further culture. Next, the medium in each well was replaced with 10% (V/V) CCK‐8 reagent, and the plates were placed in the dark for 2 hours at 37°C. Subsequently, the optical density (OD) value for each well at 450 nm was measured using the Multi‐Mode Microplate Reader (BioTek SynergyHT). Experiments were repeated independently at least three times. The IC_50_ value was calculated as the concentration of SsnB when the cell inhibition rate was 50%. Inhibition rate and cell viability were calculated using the following formulae:

$$\text{Inhibition}\;\text{rate}\;\left(\%\right)=\left[\left({\text{OD}}_{\text{control}}‐{\text{OD}}_{\text{SsnB}}\right)/\left({\text{OD}}_{\text{control}}‐{\text{OD}}_{\text{blank}}\right)\right]\times 100,$$

$$\text{Cell}\;\text{viability}\;\left(\%\right)=\left[\left({\text{OD}}_{\text{SsnB}}‐{\text{OD}}_{\text{blank}}\right)/\left({\text{OD}}_{\text{control}}‐{\text{OD}}_{\text{blank}}\right)\right]\times 100.$$

### 2.3 Colony formation assay

PCa cells in logarithmic growth stage were digested, used to prepare single‐cell suspensions and then seeded on 60 mm cell dishes at a density of 500 cells per dish. The cells were maintained in medium with different concentrations of SsnB or DMSO until each colony contained 50 cells and was visible to the naked eye for 10 days. After washing the cells with phosphate buffered saline (PBS) (HyClone), the cells on the dish surface were fixed with 4% (V/V) paraformaldehyde (Sinopharm) for 20 minutes and stained with 1% (V/V) crystal violet reagent (Solarbio) for 15 minutes. The cells were rinsed with running water and air‐dried naturally, and colonies were surveyed using an optical microscope (Olympus).

### 2.4 Wound healing assay

The effects of SsnB on the migration ability of DU145 and PC‐3 cells were evaluated by wound healing assays. When the confluence of PCa cells maintained in 6‐well plates reached 80‐90%, three wound lines were made using sterilized 200 µL pipette tips in each well. After the cells were rinsed with PBS, SsnB solutions of different concentrations were added to the FBS‐free medium for further culture. Images of the wound area at 48 hours were collected using a phase‐contrast microscope (Nikon).

### 2.5 Transwell invasion assay

Matrigel (Corning, Inc) was diluted on ice with FBS‐free medium at a ratio of 1:8. Each 24‐well invasion chamber with 8 µm pores (Corning) was covered with 100 µL of Matrigel and incubated overnight in a 37°C incubator. After DU145 and PC‐3 cells were starved with FBS‐free medium for 12 hours, they were used to prepare a suspension, which was adjusted to a density of 2.5 × 10^5^ cells/ml. Subsequently, 100 µL of the cell suspension was inoculated in the upper chamber, and 500 µL of medium containing 15% FBS and different doses of SsnB was added to the lower chamber. After incubation for 48 hours, the Matrigel and medium in the upper chamber were removed by washing with PBS and the PCa cells remaining on the membrane were swabbed with wet cotton. The invasive cells were fixed with 4% paraformaldehyde for 20 minutes and stained with 1% crystal violet reagent for another 20 minutes. Images of invasive PCa cells were obtained by a phase‐contrast microscope.

### 2.6 Annexin V‐FITC/PI apoptotic assay

The ANNEXIN V‐FITC/Propidium Iodide (PI) Apoptotic Assay Kit (Vazyme) was applied to detect the apoptotic effect of SsnB on PCa cells according to the manufacturer's protocol. DU145 and PC‐3 cells on 6‐well plates were incubated with different doses of SsnB or DMSO for 48 hours. Adherent cells were digested with trypsin and washed with ice‐cold PBS. The PCa cells suspended in 1 mL of 1× Binding Buffer provided in the kit were centrifuged at 200 × *g* for 10 minutes. The cells were re‐suspended and adjusted to a density of 1 × 10^6^ cells/mL. Then, 100 µL of the suspension in each flow tube was stained with 5 µL of Annexin V‐FITC reagent and 5 µL of PI for 10 minutes at room temperature in the dark. Finally, the cell apoptosis rate was analysed using the Guava easyCyte Flow Cytometer (Guava Technologies).

### 2.7 Cell cycle assay

Pretreated PCa cells were digested with trypsin‐EDTA and suspended in PBS. After centrifugation, the cells were incubated with 70% (V/V) ice‐cold ethanol (Sinopharm) overnight at −20°C. The cells were incubated with RNase A (Solarbio) (20 μg/mL) for 30 minutes and then stained with PI (50 μg/mL) for another 30 minutes at room temperature. Subsequently, the DNA contents of the cells were detected using the Guava easyCyte Flow Cytometer and the results were analysed using FlowJo version 10.5.3 (Tree Star).

### 2.8 Detection of reactive oxygen species generation

Reactive oxygen species (ROS) levels were detected using the permeable probe 2′,7′‐dichlorofluorescein diacetate (DCFH‐DA) (Sigma‐Aldrich Corp.), which was oxidized into fluorescent 2′,7′‐dichlorofluorescein (DCF) in the cell. PCa cells were pretreated with SsnB or DMSO in 6‐well plates. DCFH‐DA was diluted to 10 μmol/mL in FBS‐free medium. The supernatant in 6‐well plates was discarded, and 1 mL of the DCFH‐DA solution was added to each well. After incubation for 20 minutes at 37°C, DU145 and PC‐3 cells were rinsed with FBS‐free medium three times to eliminate DCFH‐DA interference in the extracellular matrix. ROS levels were observed using a fluorescence microscope and the Multi‐Mode Microplate Reader.

### 2.9 Detection of malondialdehyde, glutathione and superoxide dismutase

The Lipid Peroxidation MDA Assay Kit (Beyotime), the Micro Reduced GSH Assay Kit (Solarbio) and the Total SOD Colorimetric Assay Kit (Elabscience) were used to analyse levels of malondialdehyde (MDA), glutathione (GSH) and superoxide dismutase (SOD), respectively. All steps were performed in strict accordance with the protocols provided by the manufacturers. All levels were detected using the Multi‐Mode Microplate Reader and normalized to the protein contents.

### 2.10 Western blotting

PCa cells treated with SsnB were collected and lysed on ice with radioimmunoprecipitation assay (RIPA) lysis buffer (Beyotime) containing 1% (V/V) phosphatase inhibitor cocktail (Beyotime) and 1% (V/V) phenylmethylsulphonyl fluoride (PMSF) (Solarbio). After centrifugation, the protein concentration in the supernatant was detected using the Bicinchoninic acid (BCA) Protein Assay Kit (Beyotime). The protein samples were diluted with 5× sample loading buffer (Beyotime) to the same concentration and denatured for 5 minutes at 100°C. The protein samples were separated using the Polyacrylamide Gel Electrophoresis (PAGE) Fast Preparation Kit (EpiZyme) and transferred to a polyvinylidene difluoride (PVDF) membrane (Millipore). Next, the membranes were blocked with 5% (V/V) skim milk or 5% (V/V) bovine serum albumin (Sigma‐Aldrich Corp.) for 2 hours at room temperature. The primary antibodies included anti‐Caspase/cleaved Caspase 3 (1:1,000; ab32351; Abcam), anti‐Bax (1:4,000; 60267‐1‐Ig; Proteintech), anti‐Bcl‐2 (1:1,000; 60178‐1‐Ig; Proteintech), anti‐PI3K (1:1,000; #4292; Cell Signaling Technology), anti‐AKT (1:1,000; 10176‐2‐AP; Proteintech), anti‐phospho‐AKT (1:3,000; 66444‐1‐Ig; Proteintech) and anti‐β‐actin (1:1,000; 66009‐1‐Ig; Proteintech). The membranes were incubated with the primary antibodies overnight at 4°C and with secondary horseradish peroxidase‐conjugated antibodies (1:10,000; Zhongshan Golden Bridge Biotech) for 2 hours at room temperature. Signals were detected using the Electrochemiluminescence (ECL) Plus Kit (Wanleibio) and the Amersham Imager 680 (GE Co.).

### 2.11 Xenograft model

BALB/c nude mice (male, six‐week‐old) (Beijing Hfk Bioscience Co., Ltd.) were maintained in an SPF‐grade animal laboratory with a 12‐hours light/dark cycle at 25°C and 60‐70% relative humidity and were provided food and water ad libitum. The xenograft model was constructed according to protocols approved by the ethics committee of Shengjing Hospital of China Medical University (No. 2020PS662K). Briefly, 200 μL of 1 × 10^7^/mL PC‐3 suspension was subcutaneously injected into the axilla region of nude mice. The mice were randomly divided into a vehicle control group and an SsnB group (six mice per group). Mice in the vehicle control and the SsnB (9 mg/kg) group were injected intraperitoneally once every 2 days. Weights and tumour sizes were measured regularly using Vernier callipers. The mice were killed with euthanasia, and xenograft tumours were collected for weight measurement and subsequent experiments. The formula V = 0.5 × a × b^2^ was used to measure the volume of xenograft tumours, where the parameters a and b represent the longest and shortest diameters of the tumours, respectively.

### 2.12 Immunohistochemistry

The xenograft samples were fixed in 4% (V/V) paraformaldehyde, embedded in paraffin and cut into 3 μm sections. Immunohistochemistry was performed using the UltraSensitive^TM^ SP (Mouse/Rabbit) Immunohistochemistry Kit (Maxim), and the experimental procedures were carried out according to the instructions. The samples were incubated with the primary antibodies for Ki67 (1:500; 12202S; Cell Signaling Technology) and proliferating cell nuclear antigen (PCNA; 1:500; 10205‐2‐AP; Proteintech) overnight at 4°C, and biotinylated goat anti‐mouse/rabbit IgG secondary antibody in the kit at room temperature for 20 minutes. The samples were combined with the chromogenic diaminobenzidine (DAB; Maxim) and observed using a light microscope. ImageJ (National Institutes of Health) was used to analyse protein expression levels.

### 2.13 Statistical analyses

Image processing was performed using ImageJ, and the experimental data were evaluated by the t test, one‐way analysis of variance (ANOVA) and two‐way ANOVA using SPSS version 22.0 (SPSS) or GraphPad Prism version 8.3.0 (San Diego). All data are presented as means ± standard deviation. *P* < .05 was considered statistically significant.

## 3 RESULTS

### 3.1 SsnB reduced the proliferation, migration and invasion of PCa cells in vitro

To confirm the effect of SsnB on the proliferation of PCa cells in vitro, DU145 and PC‐3 cells were treated with SsnB at concentrations ranging from 0 μmol/L to 100 μmol/L for 48 hours and evaluated by the CCK‐8 assay. The IC_50_ concentrations of SsnB for DU145 and PC‐3 cells were 43.89 ± 3.54 μmol/L and 57.61 ± 2.24 μmol/L, respectively (Figure 1B). We subsequently selected 12.5 μmol/L, 25 μmol/L and 50 μmol/L as the concentrations for the low‐dose, medium‐dose and high‐dose groups for follow‐up experiments. Subsequently, a CCK‐8 assay was performed after treatment with various concentrations of SsnB for 24 hours, 48 hours and 72 hours. At the same point in time, cell viability in each SsnB treatment group was obviously lower than that in the control group (*P* < .05 in DU145, *P* < .01 in PC‐3). Cell viability in the high‐dose group after 24‐hours, 48‐hours and 72‐hours treatment was 73.99% ± 5.48%, 53.38% ± 7.78% and 32.36% ± 3.98% in DU145, respectively, and 76.63% ± 4.25%, 59.07% ± 5.65% and 33.77% ± 5.37% in PC‐3, respectively. In addition, the cell viability in the high‐dose group after 72‐hours treatment was significantly lower than that in the high‐dose group after 24‐hours treatment (*P* < .01 in DU145, *P* < .05 in PC‐3). The results show that the inhibitory effect of SsnB on the proliferation of DU145 and PC‐3 cells increased gradually as the concentration and treatment duration increased, indicating both concentration‐ and time‐dependent effects (Figure 1C, D). In complement with this, a colony formation assay further showed that the number of clones in the SsnB group was significantly lower than that in the control group (*P* < .05) (Figure 1E, I), which was consistent with the results of the CCK‐8 assay.

A wound healing assay was conducted to verify the effect of SsnB on the migration of DU145 and PC‐3 cells. The migration rate of DU145 in the control group, low‐dose group, medium‐dose group and high‐dose group was 78.47% ± 6.29%, 62.89% ± 4.57%, 58.88% ± 4.01% and 40.94% ± 0.72%, respectively, and that of PC‐3 in the control group, low‐dose group, medium‐dose group and high‐dose group was 62.02% ± 9.22%, 46.26% ± 4.27%, 42.66% ± 2.18% and 28.36% ± 1.28%, respectively, suggesting that the migration rate of each SsnB group was significantly decreased compared with that in the control group (*P* < .01 in DU145, *P* < .05 in PC‐3). Furthermore, the inhibitory effect on migration increased as the SsnB concentration increased (Figure 1G, H, K).

Similarly, cell invasion was significantly lower in the medium‐dose and high‐dose groups than in the control group, as determined by the Transwell assay (*P* < .01 in DU145, *P* < .05 in PC‐3). The results also indicate that SsnB can inhibit the invasion of PCa cells in a dose‐dependent manner (Figure 1F, J).

### 3.2 SsnB‐induced cell apoptosis and G2/M phase arrest in vitro

**FIGURE 2:**
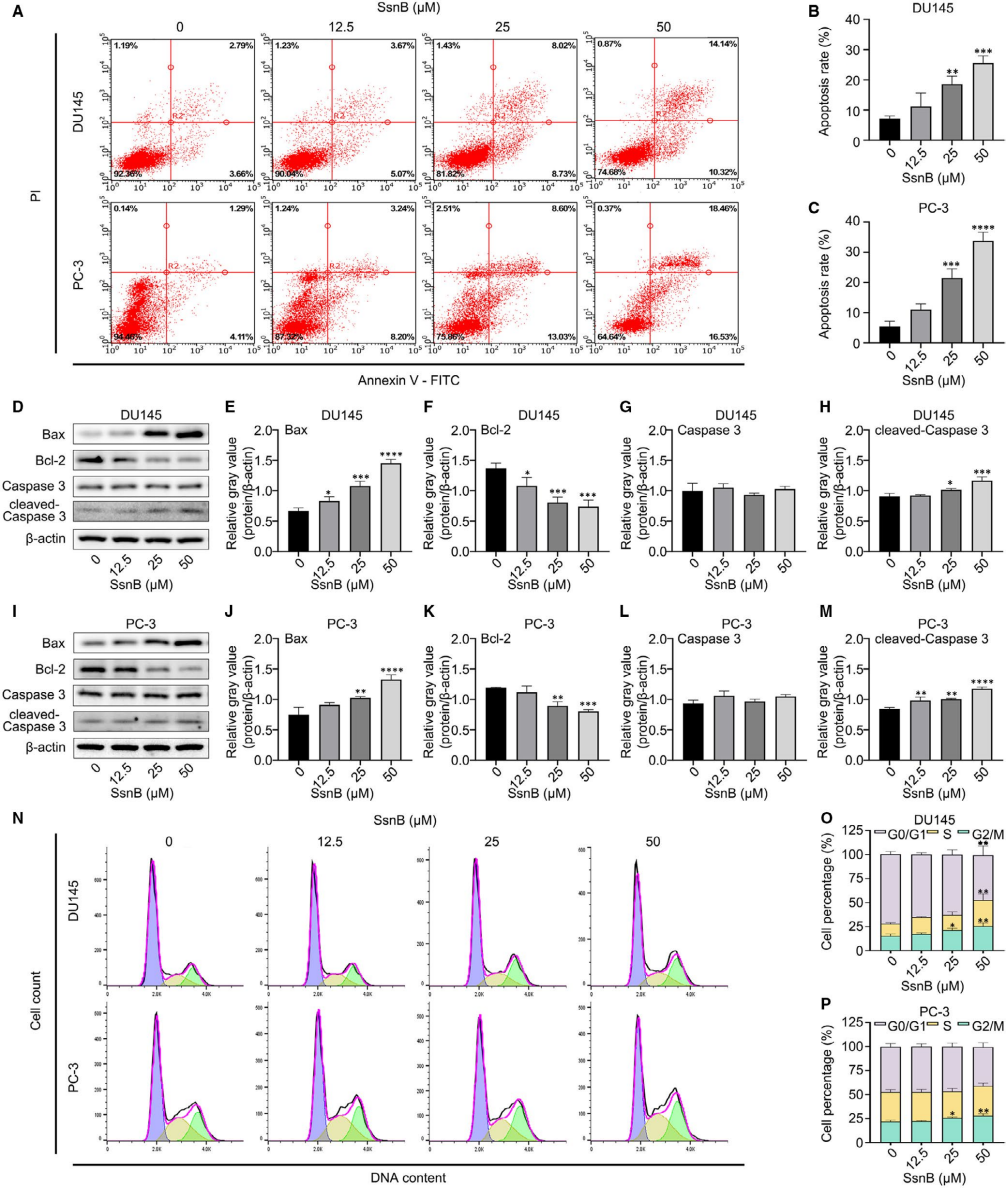
Sparstolonin B (SsnB) induces cell apoptosis and G2/M phase arrest in vitro. A‐C, Apoptosis rates of DU145 and PC‐3 in different groups, as detected by flow cytometry. D‐M, Protein expression levels of Bax, Bcl‐2, Caspase 3 and cleaved Caspase 3 in DU145 and PC‐3 cells. N‐P, Cell cycle progression detected by flow cytometry. The *X*‐axis represents the DNA content, and the *Y*‐axis represents the cell count. *P < .05, **P < .01, ***P < .001, ****P < .0001 vs the control group

As determined by Annexin V‐FITC/PI staining for 10 minutes, rates of apoptosis in PC cells treated by medium‐dose and high‐dose SsnB for 48 hours were significantly higher than that in the control group (*P* < .01 in DU145, *P* < .001 in PC‐3) (Figure 2A‐C). Moreover, a Western blot assay illustrated that SsnB treatment for 48 hours increased the protein expression levels of Bax and cleaved‐ Caspase 3 in DU145 and PC‐3, while decreasing the levels of Bcl‐2 (*P* < .05), with no change in the level of Caspase 3 (*P* > .05) (Figure 2D‐M). These findings collectively indicate that SsnB can significantly induce apoptosis in DU145 and PC‐3 cells.

As is commonly known, cell cycling is closely related to apoptosis, and the stagnation of cell cycle progression often leads to apoptosis.^17^ To determine the cause of apoptosis in PCa cells treated with 12.5, 25 and 50 μmol/L SsnB for 48 hours, flow cytometry was employed to analyse cell cycle progression. SsnB increased the proportions of DU145 and PC‐3 cells in G2/M phase (*P* < .05), accompanied by a decrease in G0/G1 phase in DU145 cells (*P* < .01) (Figure 2N–P). These results confirm that SsnB can induce apoptosis in PCa cells by G2/M phase arrest in vitro.

### 3.3 SsnB altered oxidative stress homeostasis in vitro

**FIGURE 3:**
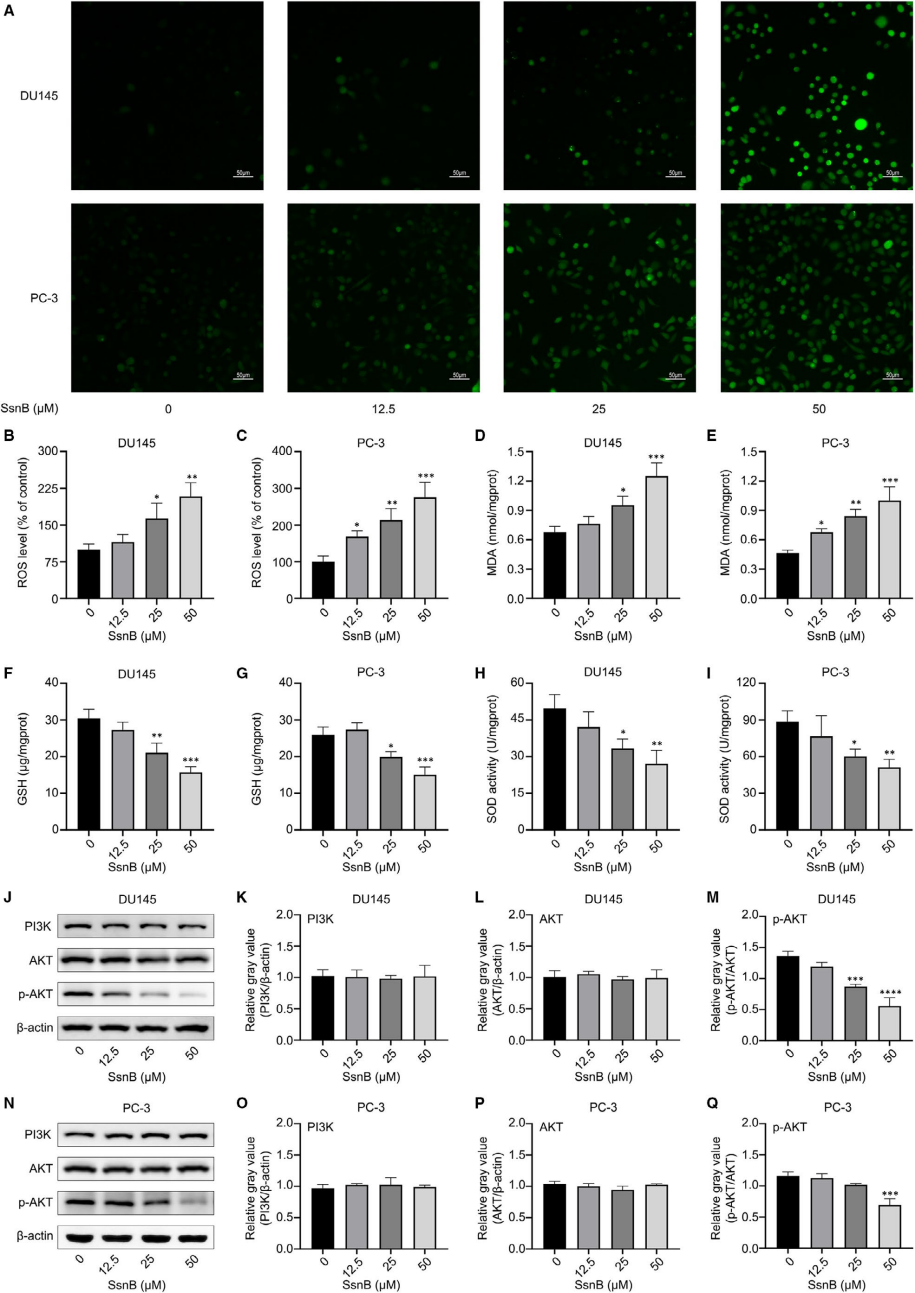
Sparstolonin B (SsnB) increases oxidative stress and suppresses the PI3K/AKT pathway in vitro. A, ROS levels of DU145 and PC‐3 detected by fluorescence microscopy (scale bar: 50 μm). The green fluorescence intensity represents the ROS level. B, C ROS levels of DU145 and PC‐3 detected using a Multi‐Mode Microplate Reader. Levels of D, E malondialdehyde (MDA), F, G glutathione (GSH) and H, I superoxide dismutase (SOD) in DU145 and PC‐3 cells detected by a Multi‐Mode Microplate Reader. J‐Q Protein expression levels of PI3K, AKT and p‐AKT in DU145 and PC‐3. *P <.05, **P <.01, ***P <.001 vs the control group

Oxidative stress contributes to the initiation and development of tumours.^18^, ^19^ Previous studies have reported that SsnB can stimulate ROS production in neuroblastoma cells.^20^ We evaluated the effect of 12.5, 25 and 50 μmol/L SsnB for 48 hours on ROS in PCa cells. As expected, green fluorescence intensities representing ROS levels in SsnB groups were significantly increased in DU145 and PC‐3 cells, based on both fluorescence microscopy (Figure 3A) and the Multi‐Mode Microplate Reader (*P* < .05) (Figure 3B, C).

In addition, the levels of MDA, GSH and SOD, related to oxidative stress, were measured. The levels of MDA (*P* < .05) were higher, while the levels of GSH (*P* < .01 in DU145, *P* < .05 in PC‐3) and SOD (*P* < .05) were significantly lower, in the SsnB groups than in the control group (Figure 3D‐I). These results suggest that SsnB can aggravate oxidative stress in PCa cells.

### 3.4 SsnB suppressed the PI3K/AKT pathway

To explore the mechanisms underlying the biological effects of different doses of SsnB treatment for 48 hours in PCa cells, Western blotting was used to measure the protein levels of PI3K, AKT and p‐AKT. The ratios of p‐AKT to AKT in SsnB groups were significantly lower than those in the control group (*P* < .001), while there were no significant differences in the levels of PI3K and AKT between groups (*P* > .05) (Figure 3J‐Q). These results suggest that the functions of SsnB are mediated by the suppression of the PI3K/AKT pathway in vitro.

### 3.5 The positive effects of SsnB on PCa cells could be partially reversed by the ROS scavenger N‐acetylcysteine (NAC)

**FIGURE 4:**
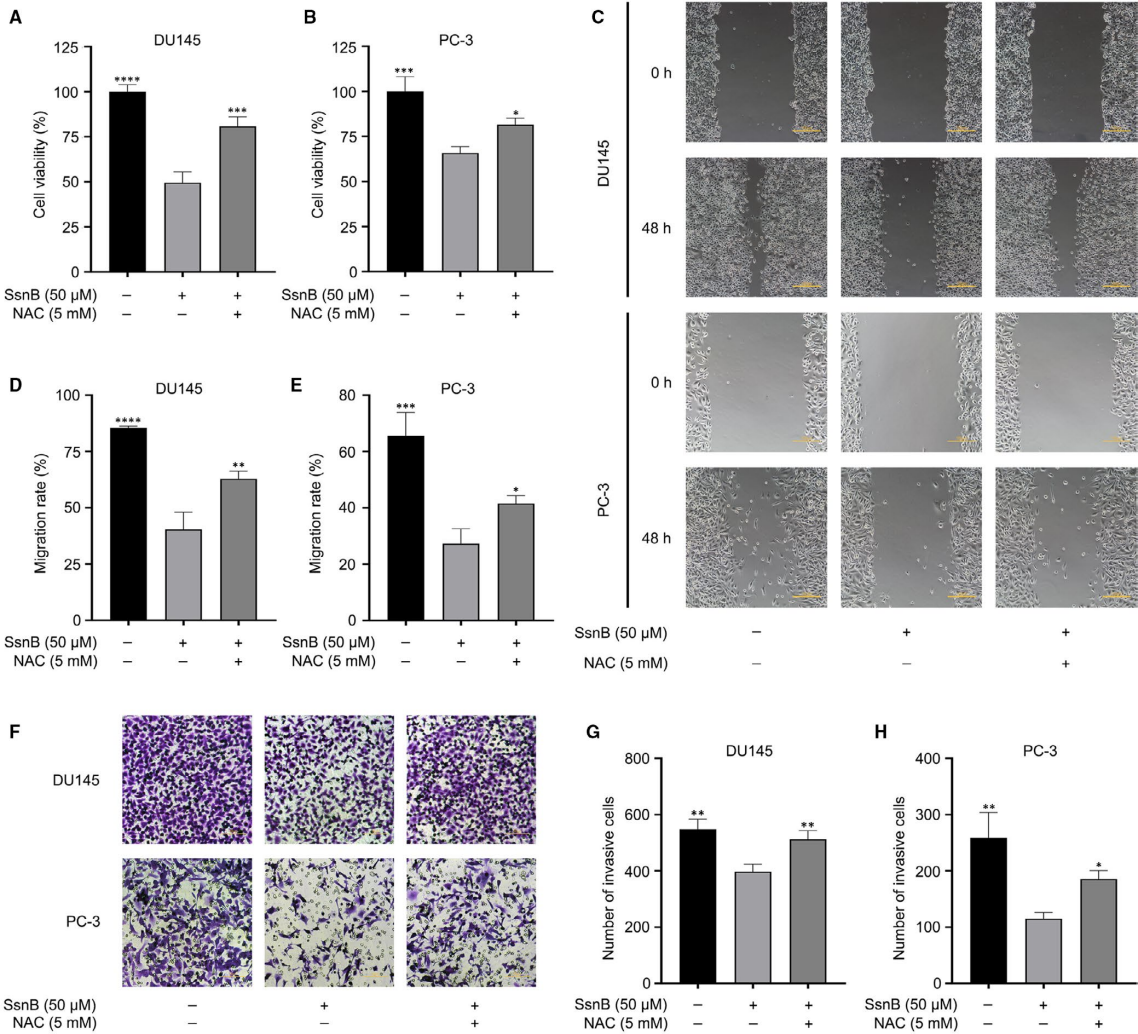
Effects of sparstolonin B (SsnB) on proliferation, migration and invasion in prostate cancer (PCa) cells could be partially reversed by the ROS scavenger *N*‐acetylcysteine (NAC). A, B Cell viabilities of DU145 and PC‐3 in different groups. C‐E, Migration abilities of DU145 and PC‐3 in different groups (scale bar: 100 μm). F‐H, Invasion abilities of DU145 and PC‐3 in different groups (scale bar: 50 μm). *P < .05, **P < .01, ***P < .001, ****P < .0001 vs the SsnB (50 μmol/L) group

**FIGURE 5:**
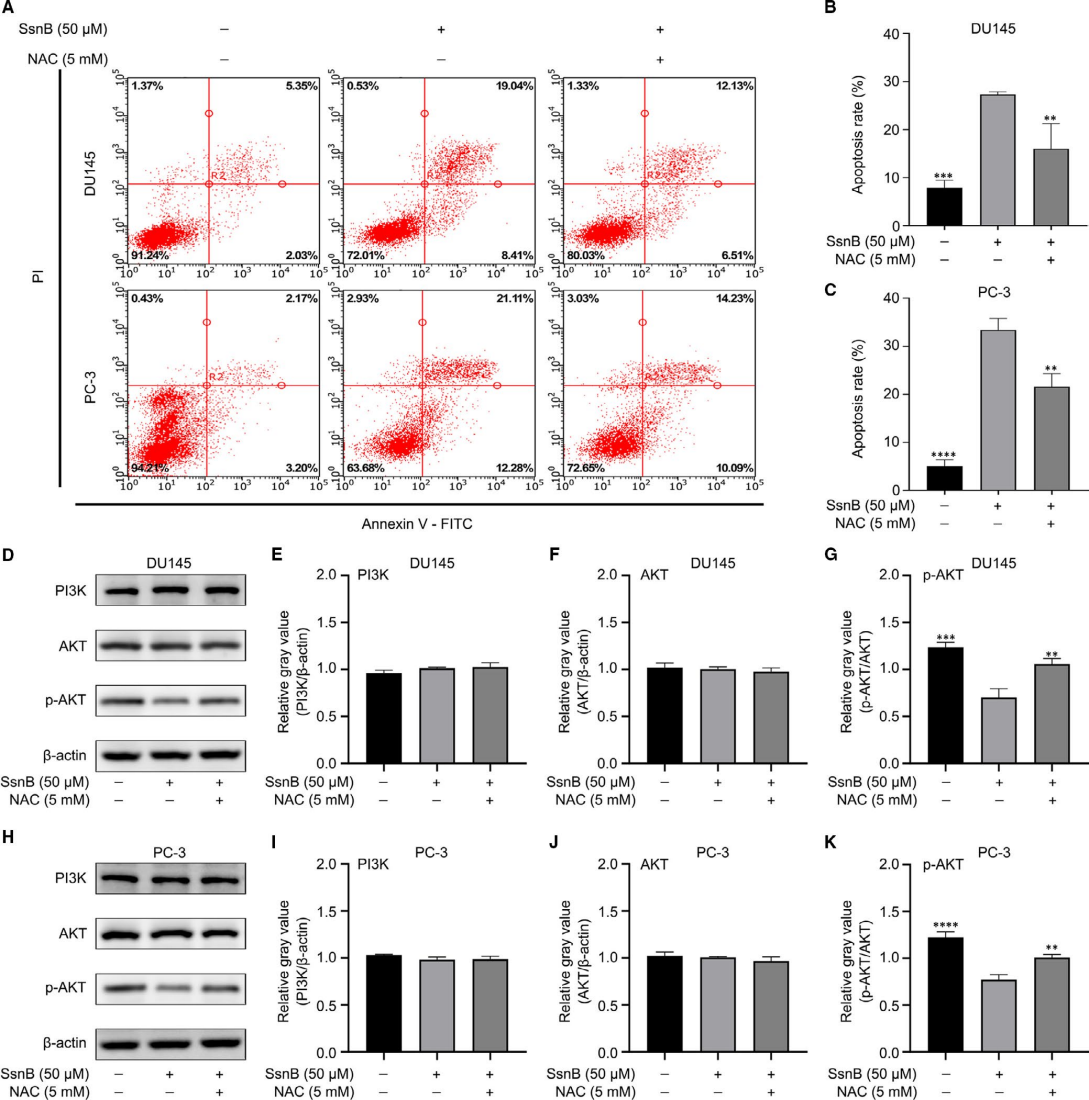
Effects of sparstolonin B (SsnB) on apoptosis and the PI3K/AKT pathway in prostate cancer (PCa) cells could be partially reversed by the ROS scavenger *N*‐acetylcysteine (NAC). A‐C Apoptosis rates of DU145 and PC‐3 in different groups. D‐K Protein expression levels of PI3K, AKT and p‐AKT in different groups. *P < .05, **P < .01, ***P < .001, ****P < .0001 vs the SsnB (50 μmol/L) group

To further investigate the relationship between oxidative stress and the effects of SsnB on PCa‐related cellular processes, the ROS scavenger NAC was used for a series of functional and pathway rescue experiments. The group treated with 50 μmol/L of SsnB and 5 mmol/L of NAC was regarded as the NAC treatment group. By means of CCK‐8 assay, wound healing assay, Transwell assay and Annexin V‐FITC/PI apoptotic assay, cell viability was higher (*P* < .001 in DU145, *P* < .05 in PC‐3) (Figure 4A, B), the wound area was larger (*P* < .01 in DU145, *P* < .05 in PC‐3) (Figure 4C‐E), cell invasion was greater (*P* < .01 in DU145, *P* < .05 in PC‐3) (Figure 4F‐H), and the apoptosis rate was lower (*P* < .01) (Figure 5A–C) in the NAC group than those in the SsnB group. These results suggest that the effects of SsnB on proliferation, migration, invasion and apoptosis in DU145 and PC‐3 cells can be partially reversed by NAC.

Similarly, Western blotting indicated that the ratios of p‐AKT to AKT in the NAC group were significantly higher than those in the SsnB group (*P* < .01), while the levels of PI3K and AKT did not differ significantly between groups (*P* > .05) (Figure 5D‐K). These results reveal that the inhibitory effect of SsnB on the PI3K/AKT pathway in PCa cells can be partially reversed by NAC. Taken together, SsnB may inhibit cellular processes in PCa by suppressing the ROS‐mediated PI3K/AKT pathway.

### 3.6 SsnB impeded the xenograft tumour growth in vivo

**FIGURE 6:**
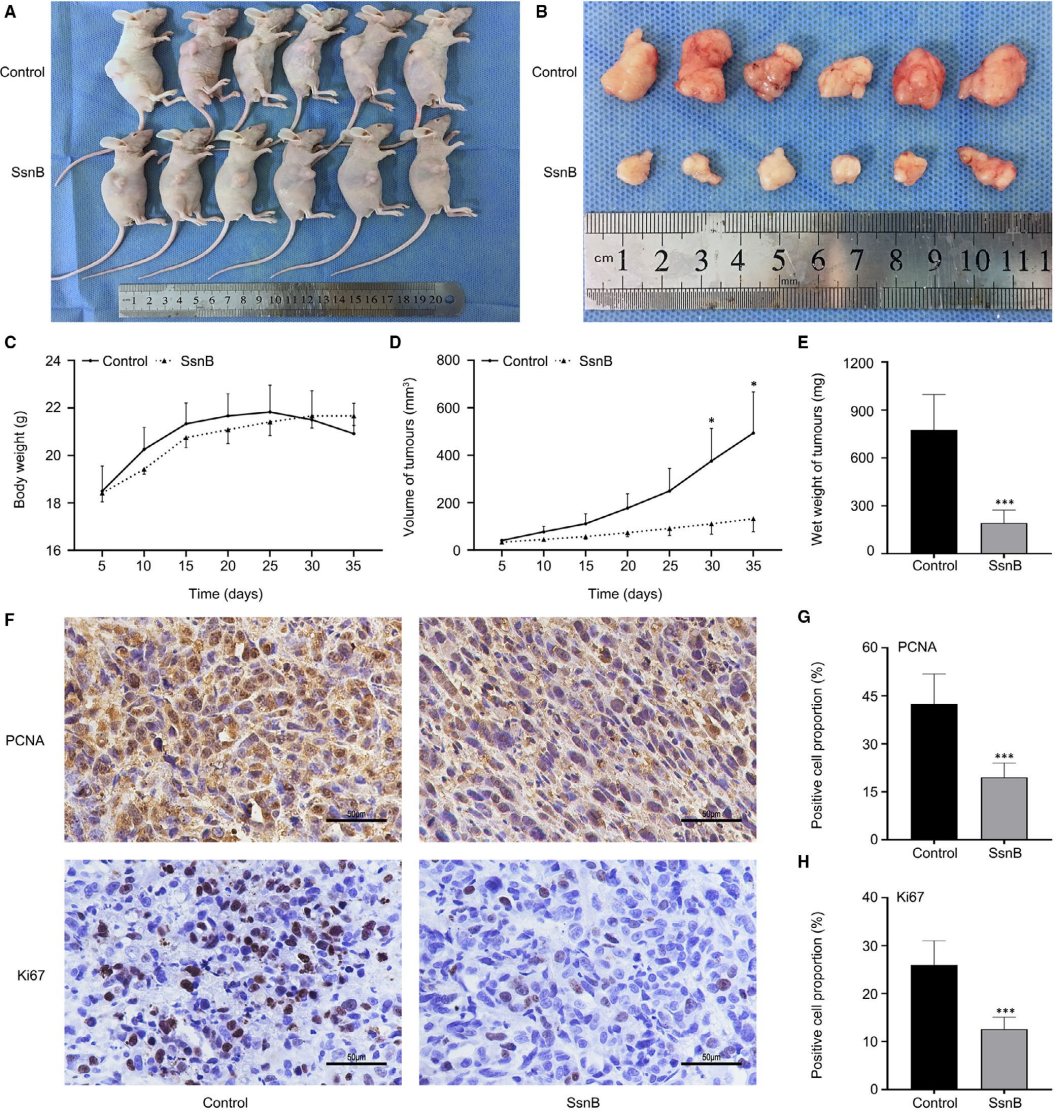
Sparstolonin B (SsnB) impeded xenograft tumour growth in vivo. A, B Xenograft tumours in nude mouse models. C, Body weights of nude mice. D, E Volumes and wet weights of xenograft tumours in different groups. F‐H, Expression levels of Ki67 and proliferating cell nuclear antigen (PCNA) in xenograft tumours detected by an immunohistochemical assay (scale bar: 50 μm). *P < .05, ***P < .001 vs the control group

To determine the role of SsnB in the growth of PCa in vivo, xenograft models were established using PC‐3 cells (Figure 6A, B). Although there was no statistically significant difference in the average body weight of nude mice between different groups (*P* > .05) (Figure 6C), the volumes of xenograft tumours in the SsnB group were significantly smaller than those in the control group (*P* < .05) (Figure 6D). Additionally, the wet weights of xenograft tumours in the SsnB group were significantly lighter than those in the control group (*P* < .001) (Figure 6E). Moreover, the expression levels of Ki67 and PCNA in the SsnB group were significantly lower than those in the control group in the immunohistochemistry assay (*P* < .001) (Figure 6F‐H). These data indicate that SsnB plays a crucial role in impeding tumour growth in vivo.

## 4 DISCUSSION

The high incidence of PCa is an important issue worldwide. Although treatments for PCa are becoming more sophisticated, rates of recurrence and metastasis remain high. TCM strategies are widely used as adjuvant therapies for PCa to relieve symptoms and prolong survival.^21^, ^22^ Despite increased and in‐depth research on TCM, these studies are still in an exploratory stage. We performed the first analysis of the effects of SsnB, a component of *S* *stoloniferum*, on PCa and the mechanisms underlying its effects.

SsnB exerts anti‐inflammatory,^23^, ^24^, ^25^, ^26^, ^27^ anti‐HIV,^28^ anti‐angiogenesis^29^ and anti‐tumour^20^, ^30^ effects in different tissues or cells. Kumar et al found that SsnB can inhibit tumour proliferation and induce apoptosis in neuroblastoma,^20^ while Tang et al proved that SsnB can inhibit migration, invasion, adhesion and metastasis in melanoma.^30^ On the one hand, the essence of SsnB is a polyphenol with two core components of xanthone and isocoumarin.^10^ Polyphenols are well known for their regulation effects on ROS production.^31^, ^32^, ^33^, ^34^ In line with this, it has been found that SsnB can promote the production of ROS in neuroblastoma,^20^ indicating that SsnB may be a kind of polyphenols with pro‐oxidant effect, which may increase the production of hydroxyl radicals through protonophoric effect and self‐oxidation to form quinones, increase ROS generation, reduce mitochondrial membrane potential and induce cell apoptosis.^35^ On the other hand, SsnB has been widely mentioned as one of toll‐like receptor (TLR) inhibitors, especially TLR‐2 and TLR‐4 in many literatures.^36^, ^37^ TLRs are believed to play a crucial role in tumourigenesis and progression,^38^ and as the upstream component, TLRs usually activate PI3K/AKT pathway to regulate the biological behaviours of tumour cells.^39^, ^40^ Furthermore, ROS could suppress the PI3K/AKT pathway.^41^, ^42^, ^43^ Based on the above, we hypothesized that SsnB can inhibit the growth of prostate cancer, possibly through the underlying mechanisms of oxidative stress and the PI3K/AKT pathway.

To verify whether SsnB had similar anti‐tumour bioactivities in PCa, we designed a series of functional experiments in vitro, including CCK‐8, clone formation, wound healing, Transwell, Annexin V‐FITC/PI and cell cycle assays. Our experimental results confirmed that SsnB can inhibit the proliferation, migration and invasion of PCa cells and induce cell apoptosis by G2/M phase arrest in vitro to exert its anti‐tumour activity. In addition, we provide the first evidence for the inhibitory effect of SsnB on PCa growth in vivo by the establishment of a subcutaneous xenograft tumour model in nude mice. The inhibitory effects were confirmed by analyses of the levels of Ki67 and PCNA in tumour slices. Based on these results of comprehensive in vitro and in vivo analyses, we demonstrate for the first time that SsnB has anti‐tumour properties in PCa.

We subsequently evaluated the mechanisms by which SsnB protects against PCa. We focused on oxidative stress, a key biological process involved in the occurrence and development of many disorders, such as cardiovascular diseases,^44^ neurological diseases,^45^ psychiatric disorders,^46^ reproductive diseases,^47^ allergic diseases^48^ and tumours.^49^ ROS can lead to DNA mutations associated with tumourigenesis, and the levels of ROS are usually elevated in tumour cells; generally, the antioxidant system of tumour cells can also be activated, to maintain stability of the redox system.^50^ It is believed that this plays a positive role in tumour treatment,^51^ and the accumulation of continuous high levels of ROS can induce tumour cell apoptosis. In clinical application, many FDA‐approved drugs also control the progression of tumours by increasing the level of ROS.^52^ Similarly, in our study, we found that SsnB increases ROS accumulation and the level of MDA and decreases the levels of the antioxidants GSH and SOD in PCa cells. Furthermore, the inhibitory effects of SsnB on the proliferation, migration and invasion of PCa cells, as well as the induction of apoptosis, could be partially restored by the addition of the ROS scavenger NAC. These results indicate that SsnB can inhibit the progression of prostate cancer by continuously producing a high level of ROS in PCa cells, disrupting the stability of the redox system and inducing oxidative stress, which is consistent with the conclusions of other studies.

The PI3K/AKT pathway is closely related to tumour‐related processes^53^ and tumour resistance.^54^ As a key pathway in PCa, it exhibits complex interactive crosstalk with other oncogenic signalling pathways.^55^ The PI3K/AKT pathway is activated by class 1A PI3Ks, and the phosphorylation of AKT results in the regulation of cellular processes.^56^ It is clear that the PI3K/AKT pathway affects cell proliferation, migration, invasion and apoptosis in PCa,^57^, ^58^, ^59^, ^60^ suggesting that the inhibition of this pathway could be a therapeutic strategy for PCa. Our experimental results showed that SsnB could inhibit the PI3K/AKT pathway and increase ROS levels, and the inhibitory effect could be partially reversed by the ROS scavenger NAC. These results indicate that SsnB can suppress the ROS‐mediated PI3K/AKT pathway.

**FIGURE 7:**
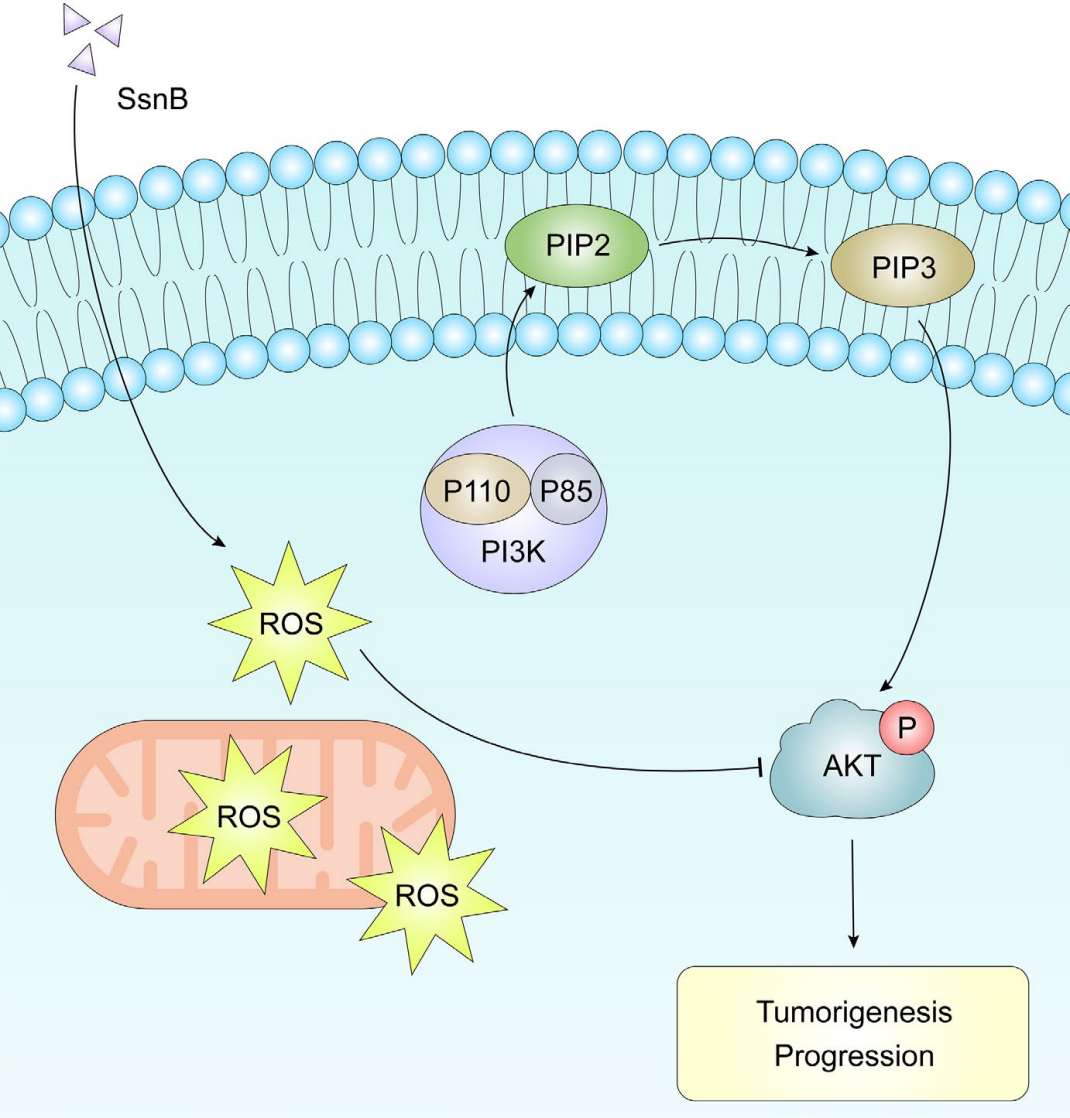
The beneficial effects of Sparstolonin B (SsnB) on tumour growth and apoptosis in prostate cancer (PCa) are mediated by the suppression of the ROS‐mediated PI3K/AKT pathway. Schematic diagram of SsnB anti‐tumour mechanism

In conclusion, we provide the first clear demonstration of the role and mechanism of action of SsnB in PCa. SsnB could inhibit the proliferation, migration and invasion of PCa cells, induce apoptosis by G2/M phase arrest in vitro and inhibit tumour growth in vivo. Furthermore, SsnB can increase ROS levels and thereby inhibit the PI3K/AKT pathway. The beneficial effects of SsnB on tumour growth and apoptosis in PCa are mediated by the suppression of the ROS‐mediated PI3K/AKT pathway (Figure 7). Hence, SsnB may be a new alternative adjuvant therapy for PCa.

## CONFLICT OF INTEREST

There are no potential conflicts of interest to declare.

## AUTHOR CONTRIBUTIONS

**Shaozhuang Liu:** Formal analysis (lead); Investigation (lead); Visualization (equal); Writing–original draft (lead). **Jiapeng Hu:** Investigation (supporting). **Changlong Shi:** Formal analysis (equal). **Li Sun:** Formal analysis (equal). **Wentao Yan:** Visualization (equal). **Yongsheng Song:** Conceptualization (lead); Supervision (lead).

## Data Availability

All data supporting the findings of our study are available from the corresponding author upon reasonable request.
